# Comparison of the presentation and electrophysiological characteristics of autoimmune nodopathies in patients with antibody-negative CIDP and CMT1

**DOI:** 10.3389/fneur.2025.1617545

**Published:** 2025-09-18

**Authors:** Changdong Song, Hengfang Liu

**Affiliations:** ^1^Henan Provincial People's Hospital, Zhengzhou, China; ^2^Department of Neurosurgery, The Fifth Affiliated Hospital of Zhengzhou University, Zhengzhou, Henan, China

**Keywords:** electrophysiology, autoimmune nodopathies, Charcot–Marie–Tooth, neurofascin, chronic inflammatory demyelinating polyneuropathy, cerebrospinal fluid protein

## Abstract

**Purpose:**

Autoimmune nodopathy (AN), as patients positive for IgG4 autoantibodies against NF155, NF186, CNTN1, or CASPR1, is a distinct form of chronic inflammatory demyelinating polyneuropathy (CIDP) that shares similar clinical and electrophysiological characteristics with Charcot–Marie–Tooth disease type 1 (CMT1). This study aimed to determine the clinical presentation and electrophysiological features of AN and compare them with antibody-negative CIDP and CMT1.

**Methods:**

We collected clinical data from 29 patients who met the European Federation of Neurological Societies/Peripheral Nerve Society (EFNS/PNS) electrophysiological diagnostic criteria for definite CIDP. Autoimmune antibodies (anti-NF155, NF186, CNTN1, and CASPR1) were tested using cell-based assays. Additionally, 17 CMT1 patients, diagnosed with hereditary motor sensory neuropathy type 1, were included. We compared the clinical and electrophysiological characteristics of AN, antibody-negative CIDP, and CMT1 patients.

**Results:**

Among the 29 CIDP patients, 10 tested positive for autoantibodies (8 for NF155, 1 for CASPR1, and 1 for CNTN1). AN patients had a younger age of onset compared to antibody-negative CIDP and were similar in age to CMT1 patients. Hand tremor was more common in AN patients (60%) compared to antibody-negative CIDP (21%) and CMT1 (5.8%). Conversely, 76.4% of CMT1 patients exhibited cavus foot, significantly higher than the 20% in AN patients. Cerebrospinal fluid (CSF) analysis revealed higher cell count and protein levels in AN patients compared to antibody-negative CIDP and CMT1. AN patients showed poor response to corticosteroids and intravenous immunoglobulin (IVIG), but rituximab was more effective. Electrophysiological findings revealed significantly prolonged distal motor latencies (DML) in the tibial posterior and peroneal nerves, as well as prolonged F-wave latencies in the ulnar and posterior tibial nerves in AN patients than antibody-negative CIDP. In contrast, compared with AN, CMT1 patients showed prolonged DML and significantly reduced motor conduction velocities (MCV) in the median and ulnar nerves. AN patients exhibited sparing of the sural nerve, whereas this phenomenon was not observed in CMT1 patients.

**Conclusion:**

In young male patients with hand tremors, demyelinating electrophysiological features (especially prolonged DML and F-wave latencies), elevated CSF protein levels, and poor response to corticosteroids, autoimmune nodopathy, AN antibody testing is recommended. Compared to AN, CMT1 patients tend to have a slower disease course, less frequent tremors, and normal CSF protein levels. A median nerve DML greater than 10 ms and MCV less than 25 m/s supports a diagnosis of CMT1.

## Introduction

Chronic inflammatory demyelinating polyradiculoneuropathy (CIDP) is an autoimmune peripheral neuropathy characterized by a variety of clinical manifestations. It is often associated with protein-cell separation in cerebrospinal fluid (CSF) analysis. CIDP typically responds well to corticosteroid therapy, but diagnosis requires the exclusion of other conditions such as POEMS syndrome, HIV-related neuropathy, and metabolic peripheral neuropathies (1).

In recent years, a special subtype of CIDP, known as autoimmune nodopathy (AN), has been identified. The concept of AN emerged after Prüss et al. (2) first proposed neurofascin 155 (NF155) as a novel immunological target in CIDP. Subsequently, additional antibodies targeting other proteins, such as neurofascin 186 (NF186), contactin 1 (CNTN1), and contactin-associated protein 1 (CASPR1), were discovered (3, 4). These proteins, together, play a crucial role in maintaining the stability of the nodal and paranodal regions of peripheral nerves, which is essential for efficient and rapid nerve conduction. However, autoantibodies targeting these proteins disrupt the integrity of the nodes of Ranvier, leading to severe abnormalities in nerve conduction, a condition now referred to as autoimmune nodopathies (AN) (5–7). Many studies have explored the clinical manifestations, antibody profiles and pathology of autoimmune nodopathy (AN). These studies have found that AN patients typically present with a younger age of onset, severe peripheral nerve demyelination, and exhibit a poor response to glucocorticoid therapy (8).

Charcot–Marie–Tooth disease type 1 (CMT1) is the most common form of CMT, accounting for approximately 50% of all cases. CMT1 is characterized by a demyelinating pathology, autosomal dominant inheritance, early onset, distal motor weakness, and moderate slowing of nerve conduction velocities (NCVs). In the early stages, distinguishing CMT1 from AN can be challenging, given their overlapping manifestations of young patients and severe demyelination (6).

This study aims to explore the clinical and electrophysiological characteristics of AN, with a particular focus on the specificity of its electrophysiological manifestations. By comparing AN with antibody-negative CIDP and CMT1, we seek to further characterize the unique features of AN and enhance the early diagnosis and treatment of this condition. Early recognition of AN is essential to avoid misdiagnosis, reduce the risk of disability, and improve patient outcomes.

## Methods

### Patients

#### Inclusion criteria

Patients who came to Henan Provincial People’s Hospital from January 2022 to January 2024, after electrophysiology and cerebrospinal fluid examination, and excluded with other peripheral neuropathies, 29 CIDP patients were enrolled. All the patients were required to meet the European Federation of Neurological Societies/Peripheral Nerve Society (EFNS/PNS) electrophysiological diagnostic criteria for definite CIDP (9), and patients who did not meet the electrophysiological standard were excluded. Patients with AN antibody (NF155, NF186, CNTN1, or CASPR1) positive were divided into AN group, and AN antibody negative were divided into antibody negative CIDP group.

Seventeen cases of CMT1 patients at the same period, confirmed through the genetic and electrophysiological evaluations were enrolled (10, 11). For patients with demyelinated CMT, the PMP22 gene was first detected by MLPA method, and then whole exome sequencing was performed. The results were verified by Sanger. In CMT1 patients, there were 13 cases of patients with repeated PMP22 mutation and 4 cases of MPZ mutation. Electrophysiological data were also collected from 22 healthy controls for comparison. These patients’ examination results were normal, and the age and gender were matched with the AN group.

#### Exclusion criteria

Patients with any of the following conditions were excluded from the study: drinking, diabetes, hyperhomocysteinemia, nephropathy, hyperthyroidism, hyperparathyroidism, lower extremity arterial thrombosis, multiple sclerosis, neuromyelitis optica, paraneoplastic syndromes, or any other diseases known to cause central or peripheral nerve damage.

Informed consent was obtained from all participants, and the study was approved by the Ethics Committee of Henan Provincial People’s Hospital, Henan Province, China (Approval No. 81873727).

CIDP patients were classified into subtypes according to the EFNS/PNS CIDP guidelines (9), which include: typical CIDP, distal acquired demyelinating symmetric neuropathy (DADS), multifocal acquired demyelinating sensory and motor neuropathy (MADSAM), and pure motor or pure sensory variants.

The clinical data collected included: sex, age at onset, age at diagnosis, disease duration, mode of onset, frequency of relapses, motor and sensory deficits, cranial nerve involvement, presence of tremor, cavus foot, Romberg syndrome, cerebrospinal fluid (CSF) cell and protein levels. In order to clarify the accurate onset age and progress of the disease course of CMT1 patients, it was necessary to make the clinical manifestations and time nodes of the patient’s weakness as detailed as possible, and to uniformly inquire about the contents, and confirm with family members to clarify the accurate time nodes.

The treatment response to corticosteroids, intravenous immunoglobulin (IVIG), and rituximab was evaluated in patients with AN and antibody-negative CIDP. Functional status was assessed using the Hughes Functional Scale (12). Patients were followed up every 3 months post-treatment. If symptoms recurred, it indicated an inadequate treatment response. Conversely, if symptoms did not recur and there was an improvement of more than one grade on the Hughes score, it was considered evidence of an effective treatment.

### Electrophysiology

Nerve conduction studies were performed on both motor and sensory nerves. During the detection, the body surface temperature was between 32 and 34°, and the stimulation volume gradually increases to reach super stimulation. For motor nerves, the median, ulnar, tibial, and peroneal nerves were assessed. For sensory nerves, the median, ulnar, and sural nerves were studied. The following electrophysiological parameters were measured: Distal motor latency (DML), Compound muscle action potential (CMAP) amplitude (distal and proximal), CMAP duration (distal and proximal), Motor nerve conduction velocity (MCV), F-wave latency, Sensory nerve action potentials (SNAP), Sensory nerve conduction velocity (SCV). DML value was detected when the distance between the stimulation point and the recording point was 7 cm on the upper limb and 9 cm on the lower limb.

To compare the degree of damage between distal and proximal nerves, the Terminal Latency Index (TLI) (13) was calculated using the formula:


$$\begin{array}{l} \mathrm{TLI}=\text{Distal conduction distance}\;(\mathrm{mm})/\\ \text{Forearm conduction velocity}\;(m/s)\times \\ \text{Distal motor latency}\;(\mathrm{ms}) \end{array}$$

Motor nerve conduction block (CB) was defined as a reduction of more than 50% in the amplitude of the proximal negative peak CMAP compared to the distal, provided the distal negative peak CMAP was ≥20% of the lower limit of normal (LLN). Probable CB was defined as a reduction of more than 30% in the amplitude of the proximal negative peak CMAP relative to the distal, excluding the posterior tibial nerve. For the Erb point (supraclavicular fossa), a reduction of more than 50% in the amplitude was considered significant (9).

Sural sparing was defined as normal sural nerve conduction with abnormal median or radial sensory nerve action potential (SNAP) amplitude, excluding cases of median neuropathy at the wrist (e.g., carpal tunnel syndrome).

Distal CMAP durations were measured in healthy controls, with the P95 values serving as the cutoff: median nerve 7.17 ms, ulnar nerve 7.74 ms, tibial nerve 6.32 ms, peroneal nerve 6.84 ms. The TLI value for the median nerve in healthy individuals was 0.36 ± 0.04, and for the ulnar nerve, it was 0.47 ± 0.03.

### Cell-based assay

Serum and CSF samples were analyzed for the presence of IgG4 autoantibodies against neurofascin 155 (NF155), neurofascin 186 (NF186), contactin 1 (CNTN1), and contactin-associated protein 1 (CASPR1) using cell-based assays (CBA). The testing was conducted by KingMed Diagnostics Medical Company.

### Statistics

Data were presented as means ± standard deviations (SD) and compared using the two-sample t-test or analysis of variance (ANOVA), as appropriate. Categorical variables were expressed as frequencies (n, %) and compared using Fisher’s exact test. A two-sided *p*-value of < 0.05 was considered statistically significant.

## Results

### Clinical presentation of antibody-positive patients in CIDP

Out of 29 CIDP patients, 10 tested positive for autoantibodies. The clinical data for these patients are summarized in Table 1. Among these, 8 patients were positive for NF155, 1 for CNTN1, and 1 for CASPR1. No patients were found to be positive for NF186.

**Table 1:** The clinical presentation of AN patients.

Patients	Sex	Age (y)	Mode of onset	MRI	NF155		Serum CASPR1	Serum CNTN1	CSF protein (g/L)
					Serum	CSF	N	N	
Patient 1	Male	15	Chronic	ND	1:1000	ND	N	N	2.39
Patient 2	Male	17	Chronic	N	1:100	ND	N	N	2.1
Patient 3	Male	36	Chronic	N	1:100	ND	N	N	1.94
Patient 4	Male	12	Chronic	Thickening of nerve roots and plexuses	1:100	1:1	N	N	2.25
Patient 5	Male	18	Chronic	ND	1:320	1:3.2	N	N	1.17
Patient 6	Male	15	Chronic	Thickening of nerve roots and plexuses	1:100	ND	N	N	2.9
Patient 7	Female	20	Subacute	ND	1:100	ND	N	N	4.8
Patient 8	Male	38	Chronic	N	ND	1:10	N	N	1.4
Patient 9	Male	59	Subacute	ND	N	ND	1:32	ND	1.43
Patient 10	Male	72	Chronic	ND	N	ND	ND	1:320	2.21

ND, not done; N, negative; CSF, cerebrospinal fluid.

Of the 8 NF155-positive patients, 7 were male and 1 was female, with an onset age range of 12–38 years. The serum antibody levels ranged from 1:100 to 1:320. Seven patients had a chronic onset, while 1 had a subacute onset. MRI of the nerve root plexus showed significant thickening in 2 patients, while no significant thickening was observed in 3 patients. All 8 patients had a poor response to hormone therapy but responded well to rituximab.

CNTN1-positive patient was a 59-year-old male who showed a positive response to corticosteroid treatment (data not shown). The CASPR1-positive patient was a 72-year-old male who did not respond to corticosteroid therapy but showed an effective response to rituximab.

### Comparison of clinical presentation in patients with AN, antibody-negative CIDP, and CMT1

The percentage of male patients in the AN group was 90%, with no statistically significant difference compared to the antibody-negative CIDP and CMT1 groups. AN patients were younger at onset than those with antibody-negative CIDP (*p* = 0.028), but their onset age was similar to that of CMT1 patients (*p* = 0.856). The average disease duration for AN patients was 10 months, which was not significantly different from the 6.5 months observed in antibody-negative CIDP patients (*p* = 0.279), but it was significantly shorter than the 91 months in CMT1 patients at presentation (*p* = 0.01) (Table 2).

**Table 2:** Clinical presentation of AN, antibody negative CIDP and CMT1 patients.

Demographics	AN (*n* = 10)	Antibody negative CIDP (*n* = 19)	CMT1 (*n* = 17)	AN vs Antibody negative CIDP *p* value	AN vs. CMT1 *p* value
Male (%)	9 (90.0)	13 (68.4)	11 (64.7)	0.271	0.148
Age at onset (year)	30.2 ± 6.5	47.7 ± 4.2	31.5 ± 16.5	0.028	0.856
Disease duration (month)	10.6 ± 9.8	6.5 ± 2.1	91 ± 112.3 (60 ± 87)	0.279	0.01
Nadir time (month)	7.8 ± 1.4	7.0 ± 1.8		0.781	
Frequency of onset	2.1 ± 0.3	1.7 ± 0.3		0.520	
Clinical phenotype (*n*, %)				0.427	
Typical	3 (30.0)	8 (42.1)			
DADS	7 (70.0)	7 (36.8)			
MADSAM		1 (5.2)			
PURE MOTOR		1 (5.2)			
PURE SENSORY		2 (10.5)			
Mode of onset (*n*, %)				0.078	0.055
Acute	0 (0)	2 (10.5)	0 (0)		
Subacute	2 (20.0)	10 (52.6)	0 (0)		
Chronic	8 (80.0)	7 (36.8)	17 (100)		
Cranial nerve involvement (*n*, %)	2 (20.0)	1 (5.2)	1 (5.8)	0.215	0.260
Limb weakness (*n*, %)				0.738	0.278
Quadriplegia	6 (60.0)	14 (73.6)	14 (82.3)		
Lower limb	3 (30.0)	4 (21.0)	3 (17.7)		
Sensory disturbance (*n*, %)				0.149	0.563
Upper and lower	5 (50.0)	5 (26.3)	5 (29.0)		
Lower limb	3 (30.0)	3 (15.7)	7 (41.2)		
Tremor (*n*, %)	6 (60.0)	4 (21.0)	1 (5.8)	0.018	0.000
Romberg (*n*, %)	3 (30.0)	8 (42.1)	5 (29.4)	0.523	0.947
Cavus foot (*n*, %)	2 (20.0)	4 (21.0)	13 (76.4)	0.094	0.004
CSF tests					
Cell (/uL)	5.7 ± 2.7	3.2 ± 2.1	2.4 ± 0.5	0.012	0.004
Protein (g/L)	2.25 ± 1.0	1.0 ± 0.6	0.3 ± 0.1	0.001	0.000
Treatment response effective (*n*, %)					
Corticosteroids	2 (25.0)	17 (100.0)		0.001	
IVIG	0 (0.0)	4 (100.0)		0.029	
Rituximab	8 (100.0)	1 (100.0)			

IVIG, intravenous immunoglobulin.

There were no significant differences between AN patients and those with antibody-negative CIDP or CMT1 in terms of cranial nerve involvement, limb weakness, sensory disturbances, or the positive rate of Romberg sign (*P1* = 0.215, 0.738, 0.149, 0.523; *P2* = 0.260, 0.278, 0.563, 0.947). However, hand tremor was more common in AN patients (60%) compared to antibody-negative CIDP (21%) and CMT1 patients (5.8%) (*P1* = 0.018, *P2* < 0.001). Cavus foot was observed in 76.4% of CMT1 patients, significantly higher than the 20% seen in AN patients (*p* = 0.004).

The cell count and protein level in the cerebrospinal fluid (CSF) of AN patients were higher than those of antibody-negative CIDP patients (*p* = 0.012, 0.001). Regarding treatment response, corticosteroids and IVIG showed poor effectiveness in AN patients, whereas antibody-negative CIDP patients had a better response to these treatments. Rituximab, however, was more effective in AN patients (Table 2).

### Comparison of electrophysiological data in patients with AN, antibody-negative CIDP, and CMT1

When comparing electrophysiological data between patients with AN and antibody-negative CIDP, AN patients demonstrated significantly prolonged DML in the posterior tibial and peroneal nerves (*p* = 0.021, 0.018). The average F-wave latencies for the ulnar and posterior tibial nerves in AN patients were 50 ms and 108 ms, respectively, both of which were longer than the 43.2 ms and 63.6 ms observed in antibody-negative CIDP patients (*p* = 0.049, 0.028). There were no statistically significant differences in motor nerve conduction between the median and ulnar nerves (Table 3).

**Table 3:** Nerve conduction in patients with AN, CIDP, and CMT.

Nerve conduction	AN (*n* = 10)	Antibody negative CIDP (*n* = 19)	CMT1 (*n* = 17)	AN vs Antibody negative CIDP *p* value	AN vs CMT1 *p* value
MOTOR					
Median nerve	10	19	17		
DML (ms)	8.2 ± 1.5	8.5 ± 4.6	11.7 ± 3.9	0.636	0.001
>6 ms	9	12	16	0.211	0.184
>8 ms	5	7	15	0.530	0.008
>10 ms	1	5	11	0.345	0.002
MCV (m/s)	36.2 ± 14.2	33.4 ± 12.9	20.2 ± 5.7	0.694	0.007
<25 m/s	3	5	12	0.711	0.024
<15 m/s	1	2	3	0.561	0.768
CMAP amplitude (mV)	5.3 ± 4.5	4.0 ± 3.0	3.6 ± 2.5	0.618	0.281
Distal CMAP duration	7.9 ± 2.0	8.9 ± 4.0	8.3 ± 2.1	0.831	0.500
CB	4	4	0	0.420	
F-wave latency (ms)	48.2 ± 7.4	53.8 ± 30.7	62.3 ± 28.2	0.953	0.029
TLI	0.27 ± 0.12	0.35 ± 0.2	0.31 ± 0.09	0.246	0.447
Ulnar nerve	10	18	17		
DML (ms)	6.2 ± 1.2	5.4 ± 1.9	8.6 ± 2.8	0.060	0.024
MCV (m/s)	30.3 ± 11.1	32.5 ± 13.0	19.4 ± 5.5	0.335	0.025
CMAP amplitude (mV)	3.8 ± 2.9	3.7 ± 2.2	3.0 ± 1.7	0.702	0.499
Distal CMAP duration	8.7 ± 1.7	8.5 ± 3.4	7.9 ± 1.6	0.299	0.931
CB	3	5	0	0.097	
F-wave latency (ms)	50.0 ± 12.5	43.2 ± 16.7	58.2 ± 16.6	0.049	0.246
TLI	0.43 ± 0.16	0.47 ± 0.15	0.47 ± 0.11	0.470	0.503
Tibial nerve	5	15	9		
DML (ms)	10.7 ± 1.4	7.6 ± 3.0^a^	9.2 ± 2.0	0.021	0.385
MCV (m/s)	35.3 ± 10.0	31.3 ± 10.0	20.4 ± 3.1	0.946	0.003
CMAP amplitude (mV)	1.6 ± 2.7	2.8 ± 3.0	0.6 ± 1.3	0.107	0.592
Distal CMAP duration	10.3 ± 2.5	9.9 ± 5.4	9.0 ± 3.3	0.272	0.828
CB	0	2	0	0.778	
F-wave latency (ms)	108.0 ± 36.3	63.6 ± 28.3	91.3 ± 20.5	0.028	0.199
Peroneal nerve	4	15	6		
DML (ms)	11.1 ± 2.4	7.3 ± 2.7	10.9 ± 1.5	0.018	0.238
MCV (m/s)	23.2 ± 10.2	29.3 ± 10.8	18.9 ± 3.6	0.107	0.833
CMAP amplitude (mV)	0.7 ± 0.2	1.6 ± 1.6	0.2 ± 0.2	0.156	0.738
Distal CMAP duration	9.1 ± 1.4	9.8 ± 5.9	6.9 ± 1.8	0.701	0.042
CB	0	5	0	0.421	
SENSORY					
Median	1	9	2	0.076	0.888
SCV (m/s)	35	44.3 ± 9.4	20.2 ± 5.3		
SNAP amplitude (uV)	10	10.2 ± 9.7	6.2 ± 0.4		
Ulnar nerve	1	9	2	0.076	0.888
SCV (m/s)	30	46.2 ± 8.0	18.9 ± 2.7		
SNAP amplitude (uV)	10	11.8 ± 8.7	5.8 ± 4.7		
Sural nerve	3	10	0	0.244	0.017
SCV	43.3 ± 2.0	44.5 ± 4.2	0		
SNAP	11.9 ± 5.4	12.6 ± 5.4	0		
Sural sparing	5	8	0	0.127	0.005

DML, distal motor latency; MCV, motor nerve conduction velocity; CMAP, compound muscle action potential; SCV, sensory nerve conduction velocity; SNAP, sensory nerve action potential; CB, conduction block; TLI, terminal latency index.

When comparing electrophysiological data between CMT1 and AN patients, the DML of the median and ulnar nerves were significantly prolonged in CMT1 patients (*p* = 0.001, 0.024). Additionally, the motor conduction velocities (MCV) of the median, ulnar, and posterior tibial nerves were significantly reduced in CMT1 patients (*p* = 0.007, 0.025, 0.003). Further analysis of the DML and MCV data for the median nerve revealed that a DML > 8 ms was more common in CMT1 patients than in AN patients (*p* = 0.008). CMT1 patients were also more likely to have an MCV between 15 m/s and 25 m/s (*p* = 0.024). CMT1 patients also exhibited prolonged F-wave latency in the median nerve (*p* = 0.025).

40% of patients with AN and 26% of antibody-negative CIDP patients showed conduction block, with the affected sites being at the elbow of ulnar nerve, forearm of median nerve, and Erb point of the ulnar and median nerve. There was no statistically significant difference in conduction block between the two groups. Notably, CMT1 patients did not exhibit conduction block.

Comparison of distal CMAP durations between the three patient groups revealed that all were longer than those of normal individuals. Above the peroneal nerve which was smaller in CMT1 than AN, there were no statistically significant differences between the three groups in other motor nerves. There were no differences in TLI values between the patient groups and normal individuals.

Sensory nerve involvement was significant in all three patient groups. Only one AN patient and two CMT1 patients had detectable sensory waveforms in the upper limbs. AN patients exhibited sparing of the sural nerve in 5 out of 10 patients during the initial examination, whereas this phenomenon was not observed in CMT1 patients (*p* = 0.005).

## Discussion

In AN patients, NF155 antibody-positive cases are the most common, typically affecting younger males with an earlier disease onset. In contrast, patients with CASPR1 and CNTN1 antibodies are generally older males and are less frequently encountered, which aligns with findings from previous studies (14, 15). The positive antibody rate in our cohort was 28.6%, which is higher than the proportion reported in earlier studies (16). This difference may be attributed to the fact that all the patients we included met strict electrophysiological diagnostic criteria for CIDP, allowing for a more focused analysis of electrophysiological differences. All AN patients exhibited elevated CSF protein levels and poor responses to conventional treatments; however, rituximab treatment showed promising efficacy. Rituximab was intravenously, 100 mg on day 1, 500 mg on day 2, and 500 mg every 6 months thereafter. Despite the frequency in the general population of AN being low, the description has been crucial to identify disease subtypes and to understand the immunopathologic mechanisms that underlie these disorders (17, 18).

Compared to antibody-negative CIDP patients, AN patients exhibited younger onset ages, more frequent limb tremors, higher CSF protein levels, and poorer responses to corticosteroid therapy. These clinical characteristics are consistent with findings from other studies (19). Previous studies have reported that recurrence rates are higher in antibody-positive patients, and their clinical recovery is slower (20). Furthermore, the titer of NF155 antibodies was found to correlate with the clinical status within individual patients but not across different patients (21, 22).

In comparison to AN patients, CMT1 patients had a longer disease duration despite similar onset ages, indicating the more slowly progressive nature of CMT1. The CSF protein levels were within normal range in CMT1 patients. Thus, early diagnosis of AN can be supported by clinical features such as the age of onset, disease duration, presence of hand tremors, cavus foot, CSF testing, and antibody analysis.20% of AN patients had cavus foot, which was related to the long course of the disease and the distal muscle atrophy of the lower limbs. Research believed that cavus foot can be seen in hereditary, acquired peripheral neuropathy, congenital, and central nervous system damage (23).

In our study, AN patients showed significantly prolonged DML in the posterior tibial and peroneal nerves, as well as prolonged F-wave latencies in the ulnar and posterior tibial nerves compared to antibody-negative CIDP. However, there were no significant differences in motor conduction velocities (MCV) or DML between the median and ulnar nerves. Kouton et al. and Ogata suggested that AN patients may have more severe involvement of DML and MCV in the median and ulnar nerves (24, 25). However, Ogata et al. found that DML and MCV were more severely affected in the ulnar nerve in AN patients, while the median nerve showed no significant difference, and F-wave latencies were prolonged in AN patients (26). Wang et al. also reported that NF155 IgG4-positive patients had significantly prolonged DML compared to serum-negative CIDP patients, with little change in MCV (27). These studies support the notion that AN patients may have more severe demyelinating damage than antibody-negative CIDP patients.

Among the eight NF155 antibody-positive patients, six had an onset age under 20 years. Two patients exhibited demyelination without CB resembling CMT1 disease. Our study revealed that CMT1 patients had more pronounced DML prolongation and MCV reduction in the median and ulnar nerves compared to AN patients. Among patients with DML > 8 ms and MCV < 25 m/s in median nerve conduction, AN accounted for 3 cases, CIDP 3 cases and CMT1 15 cases, of which 1, 2 cases and 0 case were accompanied by CB. Only 1 patient with DML > 10 ms and MCV < 25 m/s in median nerve. Unlike the conclusions of Ogata (25), they believed that MCV on median nerve <24 m/s or on ulnar nerve <26 m/s were predictive of positive antibody CIDP, while the slowest MCV not average value was selected to analysis and it had low sensitivity. We concerned that DML greater than 8 ms and MCV less than 25 m/s in the median nerve is more supportive of a diagnosis of CMT1 not AN patients. Our study suggested that most AN patients had severe demyelination than CIDP, but were less severe than CMT1, which may be related to the quick treatment, and none of the patients had a family history, and some patients had negative genetic test. For a small number of patients with AN who had severe demyelination without conduction block, further identification should be combined with lumbar puncture and family history.

It was previously believed that CMT1 was a genetic disease characterized by uniform demyelination without conduction block or waveform dispersion. However, our study found that the distal CMAP duration in CMT1 patients exhibited dispersion similar to CIDP, but no CB was observed. Manganelli et al. reported partial conduction block in proximal nerves in 4.5% of CMT1 patients (28), and Kang et al. found that both CMT1 and CIDP patients exhibited a lower ratio of proximal to distal amplitude compared to healthy controls, with CIDP showing more significant and scattered data (29). These studies suggest that both CIDP and CMT1 involve severe and heterogeneous demyelination. In CMT1 patients, secondary axonal damage at the distal motor nerve leads to waveform dispersion, which is associated with secondary demyelination of the axons.

The present study has some limitations. There were no patients positive for the NF186 antibody, Distal weakness and/or numbness was the core feature of NF186 positive patients. Sensory ataxia, tremor and central nervous system demyelination were rarely observed. Nerve conduction studies revealed predominant demyelinating with/without axonal loss (30, 31). The number of CNTN1 or CASPR1 antibody positive patient was small. Thus, increasing the sample size and multi-center research for further clinical and nerve conduction analysis is crucial. Prospective electrophysiological studies are crucial for early diagnosis of AN patients, particularly investigating the relationship between electrophysiological and clinical severity or prognosis.

In summary, our study found that AN presents with distinct clinical manifestations, high CSF protein levels and effective treatment with rituximab. Electrophysiological studies suggest more severe demyelinating damage. Compared to CMT1 patients, AN patients have short onset time, common tremors, rare cavus foot and exhibit significantly elevated CSF protein levels. The median nerve conduction can help differentiate between AN and CMT1. Analyzing AN alongside antibody-negative CIDP and hereditary CMT further underscores the unique clinical and electrophysiological features of AN, suggesting that AN represents a distinct clinical subtype within CIDP, which has important implications for its classification.

## Data Availability

The datasets presented in this study can be found in online repositories. The names of the repository/repositories and accession number(s) can be found in the article/supplementary material.
